# Prebiotics supplementation improves the endothelial dysfunction in n-3 PUFA-depleted ApoE^-/-^ mice

**DOI:** 10.1186/2049-3258-72-S1-O5

**Published:** 2014-06-06

**Authors:** Emilie Catry, Géraldine Rath, Barbara D Pachikian, Audrey M Neyrinck, Patrice D Cani, Chantal Dessy, Nathalie M Delzenne

**Affiliations:** 1 Metabolism and Nutrition Research Group, Louvain Drug Research Institute, Université catholique de Louvain, Brussels, Belgium; 2 Pole of Pharmacology and Therapeutics, Institut de Recherche Expérimentale et Clinique, Université catholique de Louvain, Brussels, Belgium

## Background

Our previous studies demonstrated that dietary n-3 polyunsaturated fatty acids (PUFA) deficiency promotes the development of non-alcoholic fatty liver disease in mice, and that modification of gut microbiota composition by prebiotics (non-digestible fructans) can improve the hepatic steatosis and serum lipids in this model [1,2]. The present study has been designed to analyze the potential involvement of prebiotic supplementation on endothelial dysfunction in n-3 PUFA-depleted ApoE knock-out mice.

## Material and methods

Wild-type (WT, n=6) and ApoE^-/-^ (KO, n=6) mice were fed with a n-3 PUFA-depleted diet for 12 weeks. Fifteen days before the end, WT (n=3) and KO (n=3) mice were supplemented with fructans as prebiotics (PRE). Second and third generation mesenteric arteries were isolated and mounted on a wire myograph. After normalization, arteries were contracted with a KCl-enriched (50mM) solution. The endothelial-dependent relaxation was evaluated after addition of increasing doses of acetylcholine.

## Results

The analysis of morphological parameters showed that mesenteric micro-arteries isolated from n-3 PUFA depleted-KO mice supplemented with PRE (KO-DEF-PRE) present an significant increasing by 20% in mean diameter and develop also an significant increasing by 35% in the basal tone compared to vessels from other groups (KO-DEF or WT-DEF). Similarly, KO-DEF-PRE micro-arteries contracted significantly more to KCl-enriched solution than vessels isolated from other groups. Finally, we measured the relaxation evoked by acetylcholine: KO-DEF-PRE micro-arteries relaxed significantly more compared to KO-DEF mice isolated micro-arteries (61.27±0.343 %KCl max vs 80.513±2.542 %KCl max, p<0.01). This effect was blunted in the presence of COX inhibitor, indomethacin.

## Conclusion

Our results suggest that fifteen days of prebiotic supplementation is sufficient to alter morphological and contractile parameters in the mesenteric bed. Importantly, prebiotic supplementation is also able to prevent the endothelial dysfunction observed in KO-DEF mice, independently of the contractile modifications. Results obtained in the presence of indomethacin appoint prostanoids as possible molecular targets, in addition to the NO/NOS pathway. Further analyses are now performed to relate changes in gut functions to cardiovascular alterations.

## Competing interests

The authors declare there is no conflict of interests.
